# Comparison of p‐Tm:YAG, TFL and Ho:YAG's in vitro ablation rates on synthetic and human stones

**DOI:** 10.1002/bco2.70067

**Published:** 2025-08-18

**Authors:** Frédéric Panthier, Alba Sierra, Etienne Xavier Keller, Marie Chicaud, Eugenio Ventimiglia, Jia‐Lun Kwok, Vincent De Coninck, Mariela Corrales, Michel Daudon, Cyril Gorny, Steeve Doizi, Laurent Berthe, Daron Smith, Olivier Traxer

**Affiliations:** ^1^ Sorbonne University GRC Urolithiasis no. 20, Tenon Hospital Paris France; ^2^ PIMM UMR 8006 CNRS‐Arts et Métiers ParisTech Paris France; ^3^ Progressive Endourological Association for Research and Leading Solutions (PEARLS) Paris France; ^4^ Department of Urology Westmoreland Street Hospital, UCLH NHS Foundation Trust London UK; ^5^ Endourology Technology Section of European Association of Urology (EAU) Arnhem The Netherlands; ^6^ Department of Urology Hospital Clinic –University of Barcelona Barcelona Spain; ^7^ Endourology & Urolithiasis Working Group Young Academic Urologists (YAU) Arnhem The Netherlands; ^8^ Department of Urology University Hospital Zurich, University of Zurich Zurich Switzerland; ^9^ Service d'Urologie CHU Limoges Limoges France; ^10^ Division of Experimental Oncology/Unit of Urology; URI IRCCS Ospedale San Raffaele Milan Italy; ^11^ Department of Urology Tan Tock Seng Hospital Singapore; ^12^ Department of Urology AZ Klina Brasschaat Belgium; ^13^ Service des Explorations fonctionnelles Hopital TENON, 4 rue de la Chine Paris France; ^14^ Endourology Academy Turkey

**Keywords:** Holmium:YAG, laser, lithotripsy, pulsed Thulium:YAG, Thulium Fibre Laser, ureteroscopy

## Abstract

**Objective:**

To compare in vitro the ablation rates of p‐Tm:YAG, TFL and Ho:YAG against synthetic and human stones.

**Material and Methods:**

p‐Tm:YAG, TFL and Low‐Power (LP) Ho:YAG were compared using 270 μm core‐diameter laser fibres (CDF); experiments with 200 μm(p‐Tm:YAG) and 150 μm‐CDF (TFL) were also included. A continuous laser emission was applied through a spiral trajectory for 20 seconds with the laser fibre tip in contact with synthetic hard (HSP) and soft stone phantoms (SSP) submerged in saline. “Dusting” settings for p‐Tm:YAG(0,6 J‐20 Hz‐Flex Long Pulse), TFL(0,5 J‐30 Hz‐Short Pulse) and Ho:YAG(0,5 J‐30 Hz‐Long Pulse) and “Fragmentation” settings for p‐Tm:YAG(1 J‐15 Hz‐Captive), TFL(1 J‐15 Hz‐Short Pulse) and Ho:YAG(1 J‐15 Hz‐Long Pulse) were analysed. Then, experiments for human calcium oxalate monohydrate (COM), uric acid (UA) and cystine (CYS) stones were performed with single laser pulses at 0.6 J, 0.8 J and 1.0 J for p‐Tm:YAG (Captive Fragmenting mode), TFL (Short Pulse) and Ho:YAG (Long Pulse). Synthetic and human stone samples were dried before three‐dimensional scanning to measure ablation rates (ARs) and ablation volume per pulse (AVP).

**Results:**

For synthetic stones with 270 μm‐CDF, the p‐Tm:YAG and TFL presented similar ARs, except in Fragmentation against HSP (95,1 ± 13,6vs67 ± 14 p = 0,02, respectively). Both p‐Tm:YAG and TFL achieved higher ARs than Ho:YAG in all settings. p‐Tm:YAG‐200 μm‐CDF and TFL‐150 μm‐CDF presented similar ARs, except in Fragmentation against HSP(78,4 ± 8vs42,5 ± 2,6 mm^3^/min,p = 0,0002). Both p‐Tm:YAG‐200 μm‐CDF and TFL‐150 μm‐CDF presented at least 50% higher ARs than 270 μm‐Ho:YAG. For human stones with COM, TFL exhibited higher AVP compared to p‐Tm:YAG and Ho:YAG across all pulse energies (258,2 ± 213vs81,7 ± 31,9vs41,5 ± 25,4 μm^3^ p = 0,01, respectively). Against UA, Ho:YAG demonstrated higher AVP compared to TFL and p‐Tm:YAG (355,2 ± 161vs99,8 ± 76,7vs292,9 ± 203,1 μm^3^ p = 0,0005, respectively). For CYS, Ho:YAG presented higher AVP but without significance (99,8 ± 76,7 vs 49,3 ± 36,3 vs 38,8 ± 12,2 μm^3^, p = 0,09).

**Conclusion:**

p‐Tm:YAG and TFL achieved higher ARs than LP‐Ho:YAG against synthetic stones in vitro. For human stones, TFL achieved the highest AVP against COM while LP‐Ho:YAG delivered higher AVPs against UA and CYS, for which TFL performed worst.

## 1 INTRODUCTION

Flexible ureteroscopy (FURS) with laser lithotripsy is now the preferred surgical option for treating urinary stones up to 2 cm in maximum diameter according to international guidelines.^1^

The Holmium:Yttrium‐Aluminium‐Garnet (Ho:YAG) laser has been the gold‐standard for endoscopic laser lithotripsy during FURS for the last twenty years, but is now joined by the Thulium Fibre Laser (TFL).^1^ Both laser sources are currently recommended for endoscopic lithotripsy.^1^ A recent meta‐analysis reported lower zero‐fragment and complication rates with TFL compared to Ho:YAG for renal stones.^2^ TFL's uniform pulse shape and low Peak Power PP(500 W) may contribute to its better efficiency during laser lithotripsy compared to Ho:YAG, which exhibits a peaked pulse shape and higher PP up to 20 000 W. Even more recently, pulsed‐Thulium:YAG (p‐Tm:YAG) technology has been proposed for endourological applications, including laser lithotripsy.^3^ p‐Tm:YAG's in vitro ability to fragment and dust stone phantoms and all human stone types has been demonstrated, as well as its safety (temperature profiles, retropulsion forces).^4^, ^5^, ^6^, ^7^ With a uniform pulse profile and an intermediate and adjustable PP, p‐Tm:YAG could overcome certain limitations of TFL, particularly its lower efficacy in fragmenting hard stones such as Cystine, where low PP may be suboptimal.^8^ Recently, an in vitro experiment demonstrated that p‐Tm:YAG and TFL exhibited higher ablation masses than Ho:YAG.^9^ However, none of these studies have compared p‐Tm:YAG, TFL and Ho:YAG lasers in one single experimental setup using ablation volumes. Indeed, ablated weight could inaccurately assess the in vitro laser ablation speed and effectiveness for both synthetic and human stones: synthetic stones are made from a mixture of powder and water that dry for 48 hours before being immersed (inducing rehydration) for lasering and subsequently dried again to measure the ablation rates.^10^ On the opposite, measuring a single laser pulse ablation weight on human stone samples may not be feasible and reproducible.

Accordingly, we aimed to compare the in vitro ablation volumes of p‐Tm:YAG, TFL and Ho:YAG, TFL on both synthetic and human stones.

## 2 MATERIAL AND METHODS

### 2.1 Pulsed‐Thulium:YAG, Thulium Fibre Laser and Holmium:YAG LASER generators

Three LASER generators were compared: a 100 W 2013 nm wavelength p‐Tm:YAG (Thulio, Dornier Medtech©, Munich), a 50 W 1940 nm wavelength TFL (50 W IPG Photonics®, Russia) and a Low‐Power (LP) 30 W 2120 nm wavelength Ho:YAG (MH1, Rocamed®, Monaco). A 270 μm single‐use core‐diameter laser fibres (CDF) were used for all laser sources, with additional experiments using 200 μm and 150 μm CDF for p‐Tm:YAG and TFL, respectively. Fibre's core diameter was verified under optical microscopy before experiments, with a 10% tolerance.

### 2.2 Stone samples

Synthetic stone phantoms and ex vivo human stones were used in our experiments. 1cm^3^ cubic Begostones (Bego©, Germany) were produced according to previously described techniques with a “powder to water” ratio of 15:3 for Hard Stone Phantoms (HSP) and 15:5 for Soft Stone Phantoms (SSP) to replicate calcium‐oxalate monohydrate and uric acid stones.^10^ Synthetic stones were dried for 48 hours at 30°C to minimize heterogeneity.

Human urinary stone with >99% pure stone composition including calcium oxalate monohydrate (COM), uric acid (UA) and cystine (CYS), were selected from an institution stone bank.^8^

### 2.3 Experimental Setup

The stone phantoms and human stones were submerged in saline solution (NaCl 0.9%, Fresenius Kabi©, France) for 30 minutes at ambient temperature before being fixed in the bench model for the experiments. The laser fibre tip was positioned vertically in perpendicular contact with the surface of the stones using a micrometric screw.

**FIGURE 1:**
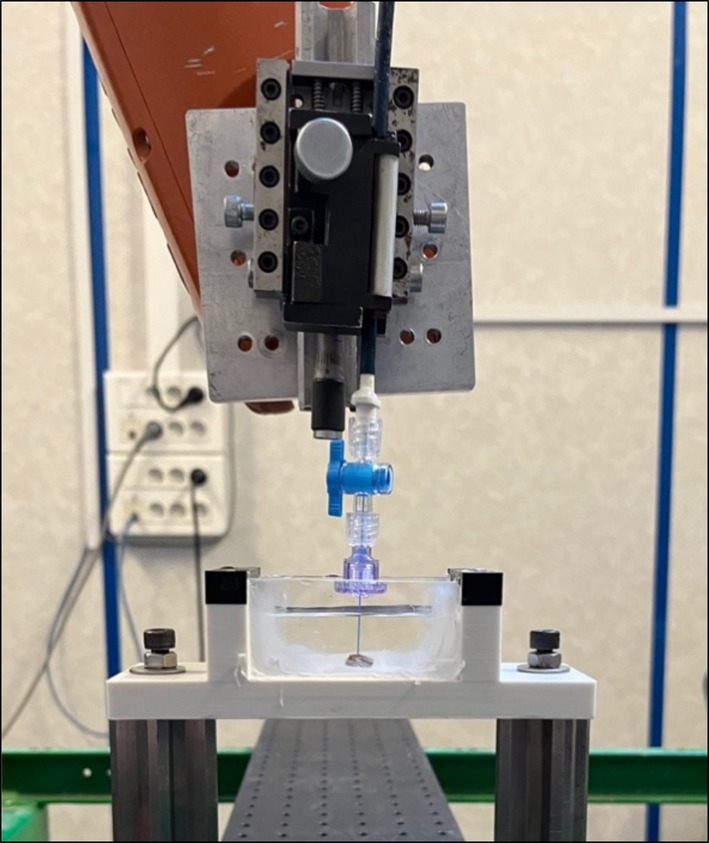
Experimental Setup. Cuvette filled with saline at ambient temperature and vertically disposed laser fibre, with a micrometric screw and fixed to a six‐axes robotic arm.

For synthetic stones, a robotic six‐axes arm (KR6R900, Kuka International©, Germany) was used to move the laser fibre along a computer‐designed Archimedean spiral trajectory of 5 mm radius with a constant distance of 1.2 mm between spiral turns for 20 seconds at a speed of 75 mm per minute. A specific support was manufactured to ensure immobility of the stone during laser emission (Figure 1). Both laser and robotic arm were started and stopped simultaneously by computer command. All tests were repeated four times.

For human stones, samples were submerged in the same experimental conditions. Single laser pulses were emitted at a suitably flat surface of each pure human stone sample to allow perpendicular energy delivery (Figure 1). This required a switch between the laser generator and the activation pedal to ensure that only a single laser pulse was delivered. Before lasing, each setup was verified by a photodiode sensor (Thorlabs©, USA) connected to an oscilloscope (InfiniiVision‐DSO5014A, Agilent‐Technologies©, France). These tests were repeated three times.

**FIGURE 2:**
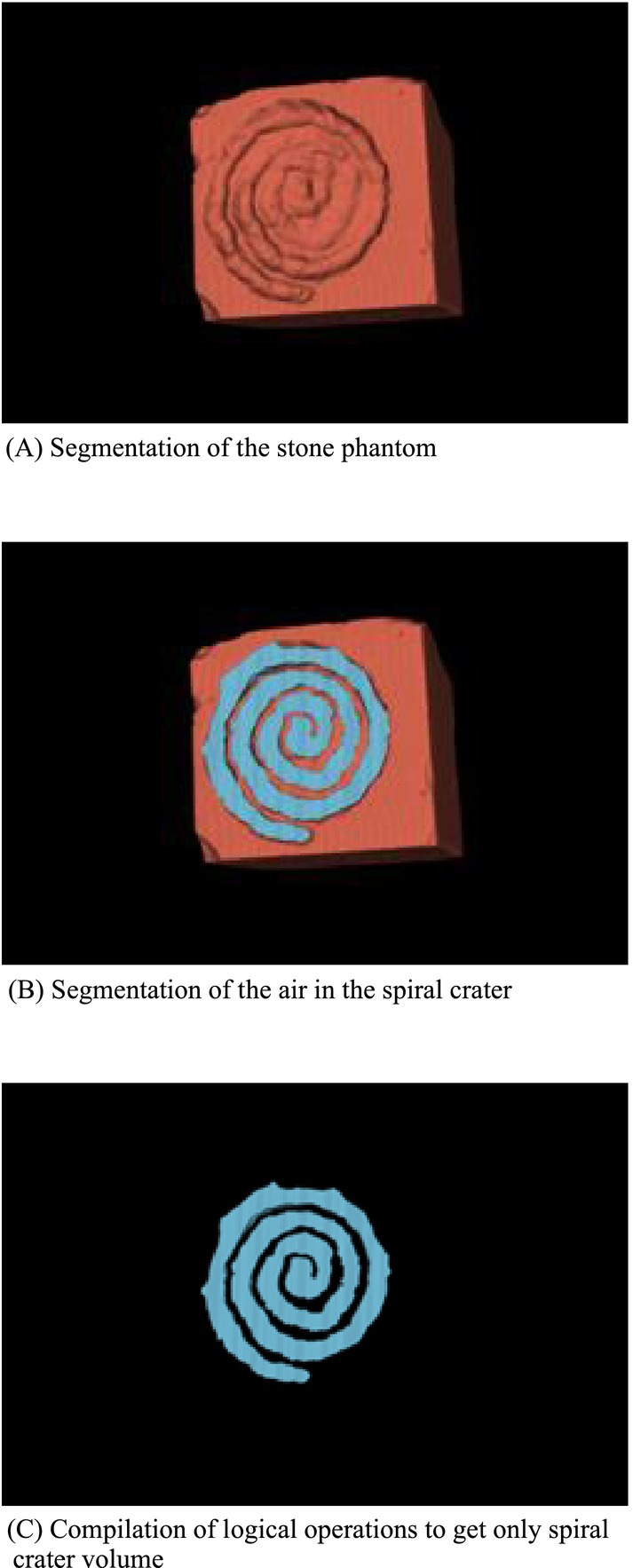
Segmentation process using 3DSlicer to assess ablation volumes based on DICOMs' non‐enhanced computed tomography. First, segmentation of the stone (a), then segmentation of the air of the spiral crater (b) and compilation of both to get the ablation volume (c).

After each experiment, Laser fibres were cleaved by ceramic scissors and stripped. Treated stones (synthetic and human) were dried for 48 hours before analysing the ablation rates (ARs) in mm^3^/min (segmented ablation volume × 3) and ablation volume per pulse (AVP) in μm^3^ using three‐dimensional scanning (micro‐CT Quantum FX, Perkin Elmer©, USA) and segmentation via 3DSlicer software (NIH©) (Figure 2).

### 2.4 LASER settings

Regarding experiments on synthetic stone phantoms, studied laser settings are listed in Table 1a. Several p‐Tm:YAG long pulse modulations were evaluated, whereas Long pulse and High Peak Power modulations were respectively selected for Ho:YAG and TFL. In Dusting, 300 and 240 J were delivered, respectively with Ho:YAG/TFL and p‐Tm:YAG, respectively. Consequently, both ARs (mm^3^/min) and normalized ablation efficiency (mm^3^/J) were evaluated. Additionally, seven p‐Tm:YAG settings combinations were investigated.

**TABLE 1a:** Holmium:YAG (Ho:YAG), Thulium Fibre Laser (TFL) and pulsed Thulium:YAG (p‐Tm:YAG) laser settings used for the in vitro experimental setup.

Laser settings	Ho:YAG		TFL		p‐Tm:YAG	
	Pulse duration (μs)	Peak Power (W)	Pulse duration (μs)	Peak Power (W)	Pulse duration (μs)	Peak Power (W)
Dusting: 0.5 J x30Hz [15 W] *(0.6 J‐20 Hz for p‐Tm:YAG)*	850	4200	1000	500	840	600
Fragmentation 1 Jx15Hz [15 W]	850	5000	2000	500	960	865
*Additional settings for p‐Tm:YAG*						

Pulse Mode	Pulse duration (μs)	Peak Power (W)	Pulse Energy (J)		Pulse rate (Hz)	
Dusting	730	565	0.5		25	
Captive	820	660	0.6		20	
	960	865	1		10	
	960	865	1		15	
Flex Long Pulse	840	600	0.6		20	
	950	888	1		10	
	950	888	1		15	

Pulse durations (μs) and Peak Powers were determined experimentally, using oscilloscopic visualization of the pulse. Ho:YAG was set on “Long Pulse” mode, TFL on “Short Pulse” and p‐Tm:YAG on “Flex Long” or “Captive” modes.

Regarding experiments on Human stone samples, laser settings are listed in Table 1b.

**TABLE 1b:** Holmium:YAG (Ho:YAG), Thulium Fibre Laser (TFL) and pulsed Thulium:YAG (p‐Tm:YAG) laser settings used for the ex vivo experimental setup.

Laser Source and Pulse Mode		Pulse Energy (J)		
**Ho:YAG (Long Pulse)**		0.6	0.8	1
**TFL (High Peak Power/Short Pulse)**		0.6	0.8	1
**p‐Tm:YAG**	**Captive**	0.6	0.8	1
	**Flex Long Pulse**	0.6	0.8	1

Pulse durations (μs) and Peak Powers (W) were determined experimentally.

For both synthetic and human stones' experiments, “low power‐low frequency” laser settings were chosen.

### 2.5 Statistical analysis

Continuous variables were reported as mean and standard deviation. Ablation experiments used Student's t‐tests, one‐way and two‐way ANOVA with Bonferroni test and multiple comparisons to compare two and more than two groups, respectively. Normalization was ensured using Shapiro–Wilk test. Two‐sided p‐values <0.05 were regarded as statistically significant.

## 3 RESULTS

### 3.1 Ablation rates for synthetic stones (Table 2, Supplementary Tables S1 and S2)

Comparing all laser sources with 270 μm‐CDF, p‐Tm:YAG presented higher AR compared to TFL and Ho:YAG against HSP in Fragmentation and Dusting (p < 0,0001) (Table 2a). Against SSP, TFL and p‐Tm:YAG presented the highest ARs in Dusting and Fragmentation (Dusting: 57,6 mm^3^/min vs 58,8 mm^3^/min vs 22,7 mm^3^/min, Fragmentation: 75mm^3^/min vs 67,4mm^3^/min vs 38mm^3^/min respectively, p = 0,001). Comparing laser sources two by two, p‐Tm:YAG and TFL presented similar ARs, except in Fragmentation against HSP, for which p‐Tm:YAG presented higher ARs (p = 0,02). Both p‐Tm:YAG and TFL presented higher ARs than Ho:YAG in all settings. Laser settings significantly influenced ARs against both HSP (p = 0,008) and SSP (p = 0,02). Similar results were reported when analysing ablation efficiencies (mm^3^/J) (Supplementary Table S1).

**TABLE 2a:** Holmium:YAG (Ho:YAG), Thulium Fibre Laser (TFL) and pulsed Thulium:YAG (p‐Tm:YAG) ablation rates (mm^3^/min) with 270 μm laser fibres, according to laser settings and type of stone phantoms.

INTERFACE	LASER SETTINGS	ABLATION RATE (mm^3^/min)						
		Ho:YAG	TFL	p‐Tm:YAG	p‐value**	Ho: YAG vs TFL*	Ho: YAG vs p‐Tm:YAG*	TFL vs p‐Tm: YAG*
HARD STONE PHANTOMS	DUSTING	16,7 ± 0,9	57,3 ± 2,4	**57,6 ± 11,7**	LASER SOURCE: **p < 0,0001**	**0,0001**	**<0,0001**	0,97
	FRAGMENTATION	32,7 ± 5,1	67 ± 14	**95,1 ± 13,6**	LASER SETTINGS: **p = 0,008**	**<0,0001**	**<0,0001**	**0,02**
SOFT STONE PHANTOMS	DUSTING	22,7 ± 1,3	**58,8 ± 5,2**	57,6 ± 8,1	LASER SOURCE: **p = 0,001**	**<0,0001**	**0,007**	0,91
	FRAGMENTATION	38 ± 6,3	67,4 ± 11,4	**75 ± 21,4**	LASER SETTINGS: **p = 0,02**	**<0,0001**	**0,005**	0,49

* Bilateral Student‐t test.

** two‐way ANOVA.

Comparing Ho:YAG with 270 μm‐CDF, TFL with 150 μm‐CDF and p‐Tm:YAG with 200 μm‐CDF, p‐Tm:YAG presented higher AR compared to TFL and Ho:YAG against HSP and SSP, except in Dusting against HSP for which TFL presented the highest ARs (p < 0,0001, and 0,02, respectively) (Table 2b). Comparing directly two laser sources, p‐Tm:YAG and TFL presented similar ARs, except in Fragmentation against HSP for which p‐Tm:YAG presented higher ARs (p = 0,0002). Both p‐Tm:YAG and TFL presented higher ARs than Ho:YAG in all settings, except for TFL versus Ho:YAG in Fragmentation against SSP (p = 0,07). Laser settings significantly influenced ARs only against HSP (p = 0,003).

**TABLE 2b:** Holmium:YAG (Ho:YAG), Thulium Fibre Laser (TFL) and pulsed Thulium:YAG (p‐Tm:YAG) ablation rates (mm^3^/min), according to the laser fibre diameter, laser settings and type of stone phantoms.

INTERFACE	LASER SETTINGS	ABLATION RATE (mm^3^/min)						
		Ho:YAG (270 μm)	TFL (150 μm)	p‐Tm:YAG (200 μm)	p‐value**	Ho: YAG vs TFL*	Ho: YAG vs p‐Tm:YAG*	TFL vs p‐Tm: YAG*
HARD STONE PHANTOMS	DUSTING	16,7 ± 0,9	**38,6 ± 4,2**	36,9 ± 10,6	LASER SOURCE: **p < 0,0001**	**<0,0001**	**0,008**	0,77
	FRAGMENTATION	32,7 ± 5,1	42,5 ± 2,6	**78,4 ± 8**	LASER SETTINGS: **p = 0,003**	**0,01**	**<0,0001**	**0,0002**
SOFT STONE PHANTOMS	DUSTING	22,7 ± 1,3	40,9 ± 6,8	**46,1 ± 3,8**	LASER SOURCE: **p = 0,02**	**0,03**	**0,005**	0,54
	FRAGMENTATION	38 ± 6,3	52 ± 13,6	**56 ± 12,7**	LASER SETTINGS: p = 0,08	0,07	**0,002**	0,64

* Bilateral Student‐t test.

** two‐way ANOVA.

p‐Tm:YAG with 270 μm‐CDF presented higher ARs compared to those with 200 μm‐CDF (significant in Captive (1 J‐10 Hz, 1 J‐15 Hz) against HSP and Flex Long Pulse (0,6 J‐20 Hz)) against SSP (Supplementary Table S2). ARs were significantly influenced by laser settings (p < 0,0001), but not by stone phantom type (p = 0,45). CDF influenced ARs only against HSP (p = 0,007).

### 3.2 Ex vivo ablation volume per pulse (Table 3)

For COM, AVPs were significantly influenced by the laser source, with the highest AVPs for p‐Tm:YAG and TFL for 0,6 J. TFL exhibited higher AVPs compared to p‐Tm:YAG and Ho:YAG at 0,8 and 1 J, respectively. AVPs significantly increased with pulse energy, despite laser source. For UA, AVPs were influenced by the laser source only at 1 J (p = 0,04). AVPs significantly increased with pulse energy, except for TFL (p = 0,26). For CYS, AVPs were again influenced by the laser source but only at 1 J (p = 0,04). AVPs increased with pulse energy only for Ho:YAG and p‐Tm:YAG, while TFL presented decreasing AVP when increasing pulse energy. Overall, AVPs were significantly influenced by the stone type, for each laser source (Ho:YAG (p < 0,001), TFL (p = 0,02) and p‐Tm:YAG (p = 0,006).

AVPs were not influenced by p‐Tm:YAG pulse modulations (Captive vs Flex Long Pulse), except against COM at 0.6 J and 0.8 J, and against UA at 1 J (Table 3b). Despite pulse modulation, AVPs increased with pulse energy. Overall, AVPs were significantly influenced by the stone type, despite pulse modulation.

## 4 DISCUSSION

### 4.1 Ablation rates and efficiency

To the best of our knowledge, this work represents the first in vitro study comparing pulsed‐Tm:YAG, TFL and Ho:YAG ablation rates using ablation volumes as the primary endpoint. Our results demonstrated similar ARs for p‐Tm:YAG and TFL on synthetic stones, which were both higher than Ho:YAG when using 270 μm‐CDF irrespective of stone phantom type (i.e. for both Hard and Soft stones). p‐Tm:YAG performed particularly well against HSP in the fragmentation setting (achieving 2.9 times higher ARs to Ho:YAG and 1.4 times higher than TFL). Ho:YAG performed least well, achieving 1.8 to 3.4 times lower ARs across the different experiments on synthetic stones.

Our results are in line with a recent in vitro comparison of p‐Tm:YAG, TFL and Ho:YAG using ablation weight in an ureter model against synthetic stones.^9^ Previously, Petzold et al demonstrated similar dusting efficiencies between p‐Tm:YAG and Ho:YAG, but at high laser fibre displacement velocity (1500 mm/min), which could be considered difficult to be extrapolated to clinical practice – in comparison, we used 75 mm/min in our study.^11^ Kraft et al reported similar loss of stone mass with p‐Tm:YAG and TFL using low frequencies (<50 Hz).^6^ However, 400 μm‐CDF were used in these studies, which are wider than most fibres used in FURS (i.e. ≤270 μm).^6^, ^11^ Our key findings align with the ablation efficiencies (mm^3^/J), showing that TFL and p‐Tm:YAG presented similar outcomes and overpassed Ho:YAG at equal settings and fibre diameter (Supplementary Table S1). Similar outcomes were also reported using the lowest available CDF with TFL (150 μm) and p‐Tm:YAG (200 μm), compared to Ho:YAG with 270 μm‐CDF. These results support the notion that pulse duration, shape and peak power are critical parameters influencing ablation efficiency—at least in controlled laboratory conditions. Notably, the p‐Tm:YAG tested pulse modes did not affect the ARs, due to their similar pulse durations (i.e. “Long Pulse”).^12^ More importantly, TFL's high water absorption coefficient compared to p‐Tm:YAG and Ho:YAG has been proposed to explain its better efficiency, but this could translate only on the adequate working distance, i.e fibre tip to stone distance, which was reported to be lower for p‐Tm:YAG compared to Ho:YAG.^13^, ^14^ These hypotheses were confirmed by Katta et al demonstrating that TFL exhibited a better efficiency in contact mode while p‐Tm:YAG performed best at 0.5 mm.^15^

Our analysis for the ex vivo human stone experiments is unique in describing single pulse AVP on human stones for p‐Tm:YAG, TFL and Ho:YAG (Table 3a).^8^, ^16^ This showed that none of the three laser sources was best in all circumstances in terms of the Ablation Volume per Pulse (AVP). Our findings on human stones also differed from the ones on synthetic stones. TFL performed best for COM stones. However, TFL performed the worst on UA and CYS at almost all evaluated pulse energy levels. There was a surprising decrease of AVP with increasing pulse energy when using TFL against CYS, consistent with the impact of peak power on laser lithotripsy, since high PP up to 4000‐5000 W could be needed to treat CYS stones, due to its specific biochemical architecture. CYS stones could also present a higher degree of heterogeneity, even if we selected only a pure sample from a local stone bank.^17^ Recently, Katta et al also reported differences in AVP with TFL and p‐Tm:YAG according to the laser source.^15^ Ho:YAG achieved the highest AVPs for UA and CYS, with AVPs two to four times higher than p‐Tm:YAG against CYS stones. p‐Tm:YAG produced AVPs in the middle‐to‐upper range for all three stone types, suggesting this technology could represent a “middle ground” compromise between TFL and Ho:YAG and with slightly better performance than TFL against CYS. Again, “Captive Fragmenting” and “Flex Long Pulse” modulations resulted in similar AVPs for COM and CYS stones, but “Flex Long Pulse” performed better on UA (Table 3b). This is consistent with their similar pulse durations/peak power.^12^ Nevertheless, we must emphasize that our results on human stones do not completely correlate with the ones on synthetic stones, supposed to reproduce COM and UA stones. The chosen powder‐to‐water ratios were selected according to Esch et al's in vitro experiments on acoustical response of synthetic or human stones, whereas the present study focused on optical interactions.^10^ Pulse modulation (at least with Ho:YAG) may influence stone ablation, but the stone composition could also be impactful, especially on the photothermal effect.^18^

**TABLE 3a:** Holmium:YAG (Ho:YAG), Thulium Fibre Laser (TFL) and pulsed Thulium:YAG (p‐Tm:YAG) ablation volume per pulse (AVP, μm^3^) on various ex vivo stone samples, using 270 μm laser fibres.

LASER SOURCE	STONE TYPE												Mean overall p‐value**
	CALCIUM OXALATE MONOHYDRATE				URIC ACID				CYSTINE				
	0,6 J	0,8	1 J	p‐value*	0,6 J	0,8 J	1 J	p‐value*	0,6 J	0,8 J	1 J	p‐value*	
**Ho:YAG**	17,8 ± 1,8	35,7 ± 7,6	71,1 ± 17,3	**0,02**	198,1 ± 44	360,1 ± 123	507,4 ± 120	**0,01**	53,8 ± 46,6	77,1 ± 90	168,4 ± 48	0,26	**<0,001**
**TFL**	58,6 ± 27,3	287 ± 109	429,1 ± 250,1	**0,05**	76,4 ± 14	147,8 ± 16	192,9 ± 25	0,26	76,3 ± 58,7	33,1 ± 3,8	35,8 ± 11,8	0,31	**0,02**
**p‐Tm:YAG**	59,2 ± 4,6	69,1 ± 24	116,8 ± 25	**0,03**	150,3 ± 19,7	178,1 ± 25,4	550,3 ± 119,3	**0,02**	28,6 ± 8,2	40,8 ± 15,1	47 ± 6,3	0,06	**0,006**
**p‐value** ***	**0,01**	**0,007**	**0,04**	‐	0,1	0,12	**0,04**	‐	0,51	0,52	**0,04**	‐	‐

* One‐way ANOVA comparing AVP according to pulse energy,

** One‐way ANOVA comparing AVP according to stone type, all pulse energies combined.

*** One‐way ANOVA comparing AVP according to laser source for each pulse energy and all pulse energies combined.

**TABLE 3b:** Thulium:YAG (p‐Tm:YAG) ablation volume per pulse (AVP, μm^3^) on various ex vivo stone samples, according to Captive and Flex Long Pulse modulations.

PULSE MODE	STONE TYPE												
	CALCIUM OXALATE MONOHYDRATE				URIC ACID				CYSTINE				Mean overall p‐value**
	0,6 J	0,8 J	1 J	p‐value*	0,6 J	0,8 J	1 J	p‐value*	0,6 J	0,8 J	1 J	p‐value*	
**CAPTIVE**	59,2 ± 4,6	69,1 ± 24	116,8 ± 25	**0,03**	121,7 ± 26,9	158,2 ± 47,6	228,6 ± 29,9	**0,005**	26,8 ± 11,1	31,8 ± 4,8	45,6 ± 2,6	0,06	**<0,0001**
**FLEX LONG PULSE**	74,8 ± 5,4	75,2 ± 24,7	105 ± 36	0,31	150,3 ± 19,7	178,1 ± 25,4	550,3 ± 119,3	**0,02**	28,6 ± 8,2	40,8 ± 15,1	47 ± 6	0,06	**0,006**
**p‐value** ***	**0,002**	**0,02**	0,26	‐	0,11	0,28	**0,02**	‐	0,64	0,37	0,59	‐	

* One‐way ANOVA comparing AVP according to pulse energy,

** One‐way ANOVA comparing AVP according to stone type, all pulse energies combined,

*** Student's t‐test comparing AVP according to pulse modulations.

### 4.2 From laboratory to clinical practice

Both Ho:YAG and TFL are recommended according to current international guidelines (European Association of Urology, Grade A).^1^ This recommendation is supported by high‐level evidence demonstrating comparable clinical efficacy and safety profiles between TFL and Ho:YAG lasers.^2^, ^19^ A recent meta‐analysis compared both technologies on stone‐free (SFR) and zero‐fragment rate (ZFR).^2^ Authors reported similar SFR and ZFR for ureteral stones. If TFL and Ho:YAG presented similar SFR for renal stones, TFL was associated with a higher ZFR than Ho:YAG (OR 3.14, 95% CI: 1.69–5.86; p < 0.001).^2^ Our results did not intend to demonstrate better success outcomes as SFR or ZFR, but focused on the ablation speed and efficiency. Those criteria may interact with SFR and ZFR, but we must emphasize our laboratory conditions. Indeed, our in vitro results were obtained in optimized and reproducible conditions, which may not fully reflect the complexities of clinical practice that may not be encountered clinically. Flexible ureteroscopy outcomes depend on multiple factors—including the surgeon's experience and technique, choice of surgical instruments, stone burden and anatomical location—none of which were included in our experimental setup, despite the use of similar parameters across laser sources. Although we observed comparable in vitro ablation rates and volumes between TFL and p‐Tm:YAG, no clinical comparative study with high‐level evidence has yet confirmed or refuted the clinical equivalence of p‐Tm:YAG. Further clinical investigations are therefore necessary before the routine use of p‐Tm:YAG in FURS can be endorsed.

As previously mentioned, the main outcomes of our experiments were ablation speed and efficiency. We acknowledge the absence of produced fragments' specific evaluation. However, only dust can be obtained from laser lithotripsy on synthetic stones because of their composition (Bego©, Germany). Regarding the human stone samples, analysing fragments after a single laser pulse would not have been feasible. A recent in vitro study included both synthetic and human stones; the synthetic ones for ablation speed and the human samples were used for dust and fragment analyses.^20^ Nevertheless, TFL and p‐Tm:YAG have demonstrated their ability to dust all kinds of human stones in previous preclinical studies.^4^, ^21^

### 4.3 Strength and limitations

Our study used a valid experimental setup for both synthetic and human stones tests, using density‐based segmentation to determine ablation volumes.^8^ That being considered, our protocol may present some limitations. First, we acknowledge that a low‐power Ho:YAG generator was included. New Ho:YAG pulse modulations including “Magneto”, “Virtual Basket”, “Vapour Tunnel” (Quanta System©, Italy) and “Moses Technology” (Boston Scientific©, USA) would have been interesting to include, resulting in different outcomes. Moreover, we used the “long pulse” mode for fragmentation setting, whereas a higher peak power (i.e. “Short Pulse”) could have shown better ablation volumes.^22^ Secondly, we recognize that a single p‐Tm:YAG was evaluated in this study (Thulio, Dornier Medtech©, Germany), whereas a second p‐Tm:YAG is nearly available on the market (Revolix HTL, Omniguide©, USA), with no published evidence regarding its pulse profile and peak power. We investigated several p‐Tm:YAG modulations, especially “long pulse” modes such as “Captive Fragmenting”, “Flex Long Pulse” and “Dusting”, because there was no available evidence on the best p‐Tm:YAG pulse modulation. p‐Tm:YAG's “short pulse” modes should be included in further evaluations. On the contrary, TFL is now preferentially used with the “High Peak Power” (500 W) mode, known to be close to the lower limit for fracturing urinary stones. Similarly, the Ho:YAG “Long Pulse” mode demonstrated similar retropulsion and ablation weights than “Moses Technology”, both outperforming “Short Pulse” mode.^23^

Thirdly, we tried to compare all laser sources with identical settings, even if this was not possible for the p‐Tm:YAG. Interestingly, in p‐Tm:YAG's “Long Pulse” modes, only “Captive Fragmenting” allows to start at low energy (≥0.6 J)‐low frequency(5‐20 Hz). At lower pulse energies, the lowest possible available frequency is 25 Hz, possibly too high to be recommended in the ureter.

Therefore, we included ablation efficiency analyses, for which our conclusions did not differ from one based on the ablation rates (Table 2a and Supplementary Table S1). Even if we emphasize that other laser settings, including higher frequencies (>30 Hz) could have been interesting, laser settings are not universal and based on individual experience.^24^ Moreover, high frequencies utilization during FURS could result in direct and indirect thermal injury, without a better efficiency or ablation speed.^20^, ^25^ The definition of dusting and fragmentation settings remains vague and has not reached a clear and precise consensus. If some authors believed that high frequency up to 80 Hz was responsible of a better dusting lithotripsy compared to low frequency (<30 Hz), Mishra et al demonstrated that low frequency was clearly associated with smaller produced fragments.^20^ Moreover, from a physical point of view, peak power and pulse energy mediate the level of stone fracture, while frequency only stands for ablation speed, as it represents the number of pulses per second. Therefore, dusting and fragmentation settings would differ among various stone compositions, as our AVP analyses suppose (Table 3). Thus, dusting and fragmentation settings were respectively defined in the present study as low(0.5/0.6 J) and high(1 J) energy, respecting low power (≤30 W) (Table 1).

Then, we acknowledge that only experimental results are presented, requiring a high volume of experiments, consuming resources and time. A better understanding of the laser‐to‐stone interaction (photo‐mechanical, photothermal or both) could allow to move towards numerical simulation. Consequently, less experiments would be required, only to confirm the defined simulation model. Further investigations should focus on this specific aspect of in vitro laser lithotripsy. Indeed, a proper optical characterization of synthetic and human stones should be conducted to define the best ratios to reproduce human stones optical properties. On the other side, we recognize that, even if we ensured that the laser fibre was in contact with the stone using a micrometric screw, a laser degradation at the fibre tip could have occurred during the 20 sec emission. Therefore, the laser fibre could have been firing in a micrometric distance mode, especially for Ho:YAG, which present the largest risk of fibre degradation compared to thulium lasers.^26^ Furthermore, the influence of the fibre displacement velocity and the chosen frequencies could have influenced our results on synthetic stones' experiments. Indeed, Aldoukhi et al demonstrated in vitro that higher fibre displacement velocities increased ablation speed for high frequencies (≥40 Hz).^27^ However, higher ablation masses were reported with TFL at low frequencies <30 Hz.^20^ Then, lasering in a crater could favour a photomechanical ablation by cavitation bubbles, as suggested by Ho et al with Ho:YAG.^28^ Finally, we acknowledge that the repetition rate in both synthetic and human stones experiment could have resulted in a certain degree of variance. Increasing the number of experiments would have limited this bias, but on one side the access to homogenous human stones samples is now limited. On the other side, 3D micro‐scanning costs should be balanced with the number of synthetic stones experiments.

## 5 CONCLUSION

p‐Tm:YAG and TFL achieved similar in vitro ablation rates and were both higher than those with Ho:YAG, using synthetic stones. With ex vivo human stones, TFL was associated with the highest ablation volume per pulse against Calcium Oxalate Monohydrate, while Ho:YAG achieved higher ablation volumes per pulse against Uric Acid and Cystine stones, for which TFL performed least well. Our study, therefore, confirms that a universal “best laser” for lithotripsy is not currently available, although the adjustable Peak Power with p‐Tm:YAG, appears to represent a technological “middle ground” between Ho:YAG and TFL, that could be useful as an “all‐rounder” for laser lithotripsy in clinical practice.

## CONFLICT OF INTEREST STATEMENT

The authors declare that they have no conflict of interest. Olivier Traxer has declared as consultant for Karl Storz, Coloplast, IPG photonics, Ambu, Quanta System and Rocamed. Steeve Doizi has declared as consultant for Boston Scientific Corporation and Coloplast. Frederic Panthier has declared as consultant for Dornier Medtech. Etienne Xavier Keller is a speaker and/or consultant for Coloplast, Olympus, Boston Scientific, Recordati, Debiopharm and Alnylam. Daron Smith has declared educational work with Olympus, Storz and Cook.

## RESEARCH INVOLVING HUMAN PARTICIPANTS OR ANIMALS

The present study was fully conducted in vitro, without human samples or participants. Therefore, this study does not involve human participants or animals but adhere to the the tenets of the Declaration of Helsinki.

## AUTHOR CONTRIBUTIONS

**F Panthier**: protocol development, data collection and management, data analysis, manuscript writing et editing. **A Sierra:** data analysis, manuscript writing et editing. **Etienne Xavier Keller:** manuscript writing et editing. **M Chicaud**: protocol development, manuscript writing et editing. **E Ventimiglia:** manuscript writing et editing. **JL Kwok:** manuscript writing et editing. **V De Coninck:** manuscript writing et editing. **M Corrales**: manuscript writing et editing. **S Doizi**: protocol development, manuscript writing et editing. **L Berthe:** protocol development, data analysis, manuscript writing et editing. **Daron Smith:** data analysis, manuscript writing et editing. **O Traxer**: protocol development, data analysis, manuscript writing et editing.

## Supporting information


**Table S1.** Holmium:YAG (Ho:YAG), Thulium Fibre Laser (TFL) and pulsed Thulium:YAG (p‐Tm:YAG) ablation efficiency (mm3/J) with 270 μm laser fibres, according to laser settings and type of stone phantoms.

**Table S2.** pulsed Thulium:YAG (p‐Tm:YAG) ablation rates (mm^3^/min), according to the laser fibre diameter, pulse mode, laser settings and type of stone phantoms.
