# Supplementary material for: Comparison of p‐Tm:YAG, TFL and Ho:YAG's in vitro ablation rates on synthetic and human stones
Source: BJUI Compass. 2025 Aug 18;6(8):e70067. doi: 10.1002/bco2.70067 (PMC12360892; doi:10.1002/bco2.70067)
Supplement: Supplementary file 1 — Table S1. Holmium:YAG (Ho:YAG), Thulium Fibre Laser (TFL) and pulsed Thulium:YAG (p‐Tm:YAG) ablation efficiency (mm3/J) with 270 μm laser fibres, according to laser settings and type of stone phantoms. [file BCO2-6-e70067-s001.docx]

| INTERFACE | LASER SETTINGS | ABLATION EFFICIENCY (mm^3^/J) | | | | | | |
| --- | --- | --- | --- | --- | --- | --- | --- | --- |
|  |  | **Ho:YAG** | **TFL** | **p-Tm:YAG** | **p-value**** | **Ho :YAG vs TFL*** | **Ho :YAG vs p-Tm:YAG*** | **TFL vs p-Tm :YAG*** |
| HARD STONE PHANTOMS | DUSTING | 0,018±0,001 | 0,068±0,009 | **0,066±0,008** | LASER SOURCE : **p<0,0001** | **0,002** | **0,001** | 0,71 |
|  | FRAGMENTATION | 0,035±0,005 | 0,074±0,001 | **0,132±0,007** | LASER SETTINGS : **p=0,003** | **0,02** | **0,0004** | **0,02** |
| SOFT STONE PHANTOMS | DUSTING | 0,0026±0,002 | 0,070±0,001 | **0,075±0,004** | LASER SOURCE : **p=0,0004** | **0,003** | **0,0005** | 0,4 |
|  | FRAGMENTATION | 0,004±0,006 | 0,074±0,001 | **0,099±0,001** | LASER SETTINGS : **p=0,005** | **0,007** | **0,0005** | 0,05 |
| **Bilateral Student-t test; **two-way ANOVA* | | | | | | | | |

**Supplementary Table 1.** Holmium:YAG (Ho:YAG), Thulium Fiber Laser (TFL) and pulsed Thulium:YAG (p-Tm:YAG) ablation efficiency (mm3/J) with 270µm laser fibers, according to laser settings and type of stone phantoms.
