# Supplementary material for: Comparison of p‐Tm:YAG, TFL and Ho:YAG's in vitro ablation rates on synthetic and human stones
Source: BJUI Compass. 2025 Aug 18;6(8):e70067. doi: 10.1002/bco2.70067 (PMC12360892; doi:10.1002/bco2.70067)
Supplement: Supplementary file 2 — Table S2. pulsed Thulium:YAG (p‐Tm:YAG) ablation rates (mm3/min), according to the laser fibre diameter, pulse mode, laser settings and type of stone phantoms. [file BCO2-6-e70067-s002.docx]

| **INTERFACE** | **LASER SETTINGS** | | | **ABLATION RATE (mm^3^/min)** | | |
| --- | --- | --- | --- | --- | --- | --- |
|  | **PULSE MODE** | **ENERGY(J)/FREQUENCY(Hz)** | | **200µm** | **270µm** | **p-value*** |
| **HARD STONE**  **PHANTOMS** | CAPTIVE | 0,6J-20Hz | | 36,9±10,6 | 48,2±5,9 | 0,14 |
|  |  | 1J-10Hz | | 29,8±4,7 | **58,2±8** | **0,02** |
|  |  | 1J-15Hz | | 56±12,7 | **95,1±13,6** | **0,003** |
|  | DUSTING | 0,5J-25Hz | | 36,1±1,9 | 44±14,9 | 0,43 |
|  | FLEX LONG PULSE | 0,6J-20Hz | | 34,3±10,4 | 57,6±11,7 | 0,1 |
|  |  | 1J-10Hz | | 46,8±13,6 | 65,1±26,2 | 0,34 |
|  |  | 1J-15Hz | | 78,4±8 | 72,2±23,7 | 0,69 |
| **SOFT STONE**  **PHANTOMS** | CAPTIVE | 0,6J-20Hz | | 46,1±3,9 | 54,3±4,1 | 0,06 |
|  |  | 1J-10Hz | | 52,3±6,6 | 40,2±6,1 | 0,08 |
|  |  | 1J-15Hz | | 64,9±10,5 | 70,6±8,7 | 0,5 |
|  | DUSTING | 0,5J-25Hz | | 35,3±7,1 | 48,8±5,8 | 0,11 |
|  | FLEX LONG PULSE | 0,6J-20Hz | | 33,6±8,7 | **57,6±8,1** | **0,02** |
|  |  | 1J-10Hz | | 39±4,2 | 53,4±11,4 | 0,1 |
|  |  | 1J-15Hz | | 62,1±15,7 | 75±21,3 | 0,4 |
| **LASER SETTINGS AND TYPE OF STONE PHANTOMS INFLUENCE ON ABLATION RATES **** | | LASER SETTINGS | | **<0,0001** | | |
|  |  | STONE PHANTOM TYPE | | 0,45 | | |
| **PULSE MODE AND LASER FIBER DIAMETER INFLUENCE ON ABLATION RATES **** | | HARD STONE PHANTOMS | PULSE MODE | 0,45 | | |
|  |  |  | LASER FIBER DIAMETER | **0,007** | | |
|  |  | SOFT STONE PHANTOMS | PULSE MODE | 0,79 | | |
|  |  |  | LASER FIBER DIAMETER | 0,08 | | |
| **Bilateral Student t-test; **two-way ANOVA* | | | | | | |

**Supplementary Table 2.** pulsed Thulium:YAG (p-Tm:YAG) ablation rates (mm^3^/min), according to the laser fiber diameter, pulse mode, laser settings and type of stone phantoms.
